# Rethreading the needle: A novel molecular index of soil health (MISH) using microbial functional genes to predict soil health management

**DOI:** 10.1371/journal.pone.0314072

**Published:** 2024-12-02

**Authors:** Heather L. Deel, Daniel K. Manter, Jennifer M. Moore

**Affiliations:** 1 United States Department of Agriculture, Agricultural Research Service, Soil Management and Sugarbeet Research Unit, Fort Collins, Colorado, United States of America; 2 United States Department of Agriculture, Agricultural Research Service, Forage Seed and Cereal Research Unit, Corvallis, Oregon, United States of America; National Taiwan University, TAIWAN

## Abstract

Soil health relies on the actions and interactions of an abundant and diverse biological community. Current soil health assessments rely heavily on a suite of soil biological, chemical, and physical indicators, often excluding molecular information. Soil health is critical for sustainable agricultural production, and a comprehensive understanding of how microbial communities provide ecosystem services can help guide management practices. To explore the role of microbial function in soil health, 536 soil samples were collected from 26 U.S. states, representing 52 different crops and grazing lands, and analyzed for various soil health indicators. The bacterial functional profile was characterized using 16S ribosomal RNA gene sequencing paired with PICRUSt2 to predict metagenome functions. Functional data were used as predictors in eXtreme Gradient Boosting (XGBoost), a powerful machine learning algorithm, and enzymes important to soil health indicators were compiled into a Molecular Index of Soil Health (MISH). The overall MISH score significantly correlated with non-molecular measures of soil health and management practice adoption. Additionally, several new enzymes were identified as potential targets to better understand microbial mediation of soil health. This low-cost, DNA-based approach to measuring soil health is robust and generalizable across climates.

## Introduction

Jenkinson [1] described the microbial community as the “eye of the needle through which all nutrients pass”. This pioneering work launched a new research emphasis to link soil microbes with key soil functions important for plant productivity and ecosystem health. With rapid advances in sequencing technologies using marker genes, numerous studies have linked shifts in the soil microbial community with land use [2, 3] and fundamental functions of healthy soils [4–8], including nutrient cycling [9], aggregate stability [10], carbon sequestration [11], and plant health and crop productivity through plant growth-promoting bacteria [12, 13]. Additionally, the microbiome provides defenses against environmental stresses like disease [14], drought [15–17], and flooding [18]. These marker gene studies quantified microbial community composition metrics (e.g., alpha and beta diversity) and/or relative abundances of specific taxa. While information about the actions and interactions of microbes holds great promise to support future developments in sustainable agriculture [19], interpretations are hampered. A significant limitation of taxonomy is a decoupling of microbial function from composition such that the composition may change in response to external perturbations while function does not [20]. This is an example of functional redundancy, or the ability of a broad range of taxa to perform similar metabolic functions [20, 21]. Consequently, shifts in community composition do not provide answers as to why or how the community function changes. This is exacerbated in large-scale studies in which community composition is more likely to differ by region even when function is similar [20]. Conversely, microbiomes are capable of changing function without changing composition, as was shown in Bowles et al. [22] in which enzyme activities changed more dramatically than the soil taxonomic community under different nutrient sources and rates. These caveats highlight the importance of characterizing function rather than taxonomy for a more complete understanding of microbial responses to the environment.

Soil enzymes are critical catalysts responsible for biochemical reactions necessary to support soil life and numerous ecosystem functions. Enzymes that are known to differ across land use management strategies and disease states include (but are not limited to) carbohydrate hydrolases (e.g., β-glucosidase, β-N-acetylglucosaminidase, chitinase, catalase, invertase etc.), sulfur cycling enzymes (e.g., arylsulfatase), phosphorus cycling enzymes (e.g., phosomonoesterases, phosphodiesterase), and nitrogen cycling enzymes (e.g., amidohydrolases and enzymes involved in ammonia oxidation, protein decomposition, denitrification, and nitrogen fixation) [23–25]. These enzymes have been studied for decades and are commonly measured because they are closely linked with nutrient cycling and mineralization. However, most soil enzyme assays are conducted using bench-scale approaches where a limited number of enzymes are evaluated. Furthermore, microbes can produce at least 2500 different enzymes [26]. Thus, a broader and non-specific approach is necessary to effectively capture the diverse microbial functions that collectively enhance soil health.

The appropriate methodology for assessment of functional gene profiles is not without controversy with recommendations ranging from biochemical to molecular techniques. Although biochemical enzymatic and targeted molecular techniques (e.g., gene-specific quantitative PCR) can provide information on how microbial communities respond to management and climate [27], these approaches require a known substrate and an individual assay for each target enzyme or gene [28]. For more inclusive molecular techniques, two widely available options are whole genome sequencing [29] and metagenome prediction tools (e.g., PICRUSt2 [30], Tax4Fun [31]) using phylogenetic reconstruction. Sun et al. [29] compared metagenome prediction tools (PICRUSt, PICRUSt2, Tax4Fun) with whole genome sequencing for a variety of sample types. Overall, they found very high spearman correlations (r > 0.622) between gene relative abundances; however, significant differences between groups were more consistent with samples of less complexity (e.g., human metagenomes) versus higher complexity (e.g., soil metagenomes). Furthermore, Rodriguez and Konstantinidis [32] estimated that soil samples require a 100-fold or more greater sequencing depth to achieve 95% coverage for a single soil (50 Gbp) vs human (0.5 Gbp) metagenome sample. To achieve this level of coverage, a single soil sample would require upwards of four Illumina MiSeq (~15 Gbp) sequencing runs or one Oxford Nanopore (~48 Gbp) sequencing run per sample. Assuming a consumable cost of $1,000 per Oxford Nanopore sequencing run, metagenome sequencing of 500 samples at 95% coverage would cost approximately $500,000. In contrast, gene (e.g., 16S rRNA) sequencing costs approximately $20–50 per sample [33]. Assuming a consumable cost of $30 per sample, a 500-sample study using amplicon sequencing and phylogenetic reconstruction would cost $15,000. For wide-scale surveys, such as the one conducted here, whole genome sequencing efforts for complex soil samples are currently cost-prohibitive and unfeasible.

As described above and outlined in Manter et al. [4], many studies have identified specific taxa or key functional genes that respond to management practices and are associated with healthy soils. More recently, machine learning techniques have been used to align specific taxa as predictors of traditional soil health indicators [7], but we are unaware of any studies that have developed a microbial functional index that represents their collective contribution to soil health. Thus, our objective was to develop and test a new soil health index based on the molecular characterization of microbial functional capacity. To tackle this complex goal, we sequenced over 500 soil samples previously used as part of a national soil health assessment [34] and posed two interdependent questions: 1) Can the relative abundance of enzymes as estimated using PICRUSt2 predict individual soil health indicators using a random forest modeling approach? 2) Can we use this model to develop a new molecular index of soil health (MISH) that is sensitive to management at a national scale? Previous comparisons between gene abundances derived from PICRUSt2 phylogenetic reconstruction and qPCR have also been shown to be significantly correlated [35] but may be influenced by primer-specificity. Since our goal was to assess the entire microbial functional capacity in soil samples and develop an untargeted molecular index of soil health, we utilized 16S rRNA amplicon sequencing and gene abundance estimates from PICRUSt2 as both the most comprehensive and cost-effective approach currently amenable to a large-scale national survey of soils.

## Methods

### Sample collection and DNA sequencing

Details on soil collection, management histories, geography, and soil health measurements are provided in [34]. Briefly, subsamples from the 536 soil samples (0–15 cm) collected from 26 states in the U.S. representing annual cropland (n = 335), perennial cropland (n = 91), and rangeland (n = 110) systems were frozen and shipped to the U.S. Department of Agriculture, Agricultural Research Service, Fort Collins, CO. DNA extraction, PCR amplification, and library preparation were conducted following protocols commonly used in our laboratory [36]. Briefly, DNA was extracted from 0.25 g subsamples using the Qiagen DNeasy Powersoil Pro Kit (Qiagen, Germantown, MD) using a 10-min vortex lysis step and a fully automated Qiagen QIAcube robot. DNA quality was assessed using a Nanodrop 1000 (Thermo Scientific, Waltham, MA) and quantified fluorometrically with the Invitrogen dsDNA HS Assay Kit on a Qubit 2.0 (Life Technologies, Carlsbad, CA). The V3-V4 hypervariable region of the 16S rRNA gene was amplified and prepared for sequencing using the Illumina MiSeq Reagent Kit v3 using the following primers: forward 5′-*TCGTCGGCAGCGTCAGATGTGTATAAGAGACAG*CCTACGGGNGGCWGCAG-3′ and reverse 5′-*GTCTCGTGGGCTCGGAGATGTGTATAAGAGACAG*GACTACHVGGGTATCTAATCC-3′ with Illumina adapter sequences denoted in italics and underlined. The master mix consisted of 2 μL sample genomic DNA, 10 μL of 2X Maxima SYBR Green (Thermo Scientific, Waltham, MA, USA), and 2 μL each (10 μM) of forward and reverse primers for a total 20 μL reaction mix. The PCR thermal cycling conditions were as follows: 95°C for 5 min, 30 cycles of 95°C for 40 s, 55°C for 120 s, 72°C for 60 s, and a final annealing at 72°C for 7 min. The resulting amplicons were purified using an in-house preparation of solid phase reversible immobilization (SPRI) magnetic beads.

Samples were barcoded using Illumina Nextera XT index sequences added by a second PCR amplification. The master mix (50 μL) consisted of 5 μL of first-round PCR product, 25 μL of 2X Maxima SYBR Green (Thermo Scientific, Waltham, MA, USA), 10 μL water, and 5 μL each of forward and reverse indices. PCR reactions were amplified at 95°C for 3 min, 8 cycles of 95°C for 30 s, 55°C for 30 s and 72°C for 30 s, followed by final annealing of 72°C for 5 min. Following amplification, the PCR product was cleaned using SPRI beads and quantified using a Qubit fluorometer (Thermo Scientific, Waltham, MA, USA). Final library size and purity were verified using a TapeStation system (Agilent Technologies, Santa Clara, CA, USA) and the Kapa Biosystems kit (Sigma Aldrich, St Louis, MO, USA). The final pooled sample was diluted to 4 nM with ddH_2_O, denatured with 0.2 N NaOH, and a final dilution to 15 pM with HT1 buffer. Sequencing was performed on an Illumina MiSeq using the v3 600 cycle kit (Illumina, San Diego, USA) with a 25% PhiX spike-in control. DNA sequence processing consisted of primer removal from demultiplexed raw fastq files using Cutadapt v3.2 [37] and inference of amplicon sequence variants using the default pipeline in DADA2 [38]. All sequence variants were classified using the default NCBI-linked 16S rRNA reference database available from Emu v3.0.0 (https://github.com/treangenlab/emu) using minimap2 v2.22 [39].

### Functional profiling

The bacterial community functional profiles were created using the metagenome prediction pipeline, PICRUSt2 [30]. The full pipeline (picrust2_pipeline.py) was used with the representative sequences and biom tables for each sequencing with the “—stratified” (to create stratified tables at all steps) and “—skip_norm” (to skip normalizing sequence abundances by predicted marker gene copy numbers) parameters. Additionally, hidden state prediction (hsp.py) was used to predict 16S copy numbers with the “-n” parameter so that Nearest-sequenced taxon index (NSTI) values were calculated.

The stratified metagenome output (pred_metagenome_contrib.tsv.gz) and the predicted 16S copy numbers (marker_predicted_and_nsti.tsv.gz) were imported into R. The predicted 16S copy numbers were used to correct bacterial abundances, and relative abundances were calculated using the general equation taxon_relative_abundance / 100 * genome_function_count / genome_16S_count. Functional gene relative abundances were calculated for each gene associated with an enzyme commission number (EC), then converted to a feature table of ECs per sample and merged with metadata.

### Previous indicator ratings and management indices used for model development and testing

We developed and tested our new molecular-based index against the three soil health metrics from our previous national assessment: 1) individual soil health indicator ratings; 2) an overall soil health index; and 3) our Soil Health Management Index. The first two were developed using a structural equation model (SEM) that accounted for differences in climate and texture, thus enabling comparison at a national level, and are described in detail in Deel et al. [34]. Briefly, indicator ratings were calculated using the embedded SEM regressions to predict indicator values at each location based on clay content and climate zone, and the residuals (observed–predicted) were converted into a rating using the empirical distribution function in R (S1 Fig). Soil health indicator ratings were calculated for two physical properties (wet aggregate stability [AggStab], available water capacity [WaterCap]) and four biological properties (soil organic matter [SOM], active carbon [ActiveC], autoclaved-citrate extractable protein [ACE], and soil respiration [Resp]). Details of all protocols for the soil health indicators are provided by Schindelbeck and Moebius-Clune [40]. These indicators are among those that have been evaluated for use in “standardized, rapid, and quantitative assessments of soil health based on relevance to key soil processes [and] response to management” [41]. In addition, this SEM combined the contribution of each soil health indicator into a single latent variable of soil health, which we refer to as SEMWISE (Structural Equation Model for Well-Informed Soil Evaluation) [34]. Similar to the individual ratings, we used the empirical distribution function in R to transform these values into an overall SEMWISE rating that ranged from 0–100. The overall ratings were then grouped into five equidistant bins (very low: 0–20, low: 20–40, med: 40–60, high: 60–80, and very high: 80–100) to reflect soil health status across the samples.

Our Soil Health Management Index (SHMI) was designed to translate the influence of multiple management practices into a single index based on three soil health principles [34]. The practices include increasing plant biodiversity, minimizing soil disturbance, and maximizing living roots and soil cover. The SHMI score is then grouped into five bins, with lower values representing management systems of low soil health (e.g., monocultures with intensive tillage practices) and higher values approaching a management system represented by all soil health principles (e.g., perennial grazing lands with a diversity of plant species or cover crops with no-till and/or diverse crop rotations). Distilling management into a single index allows for the comparison of management across a wide range of agricultural systems that differ in their management histories and captures the influence of multiple practices (e.g., cover cropping and tillage).

### Molecular index development and testing

All analyses were performed in R v4.4.0 [42]. We used Extreme Gradient Boosting decision trees (XGBoost) to model the relationship between microbial functional gene profiles (i.e., EC relative abundances) and the SEMWISE-derived soil health indicator ratings. XGBoost has been shown to perform well on microbiome data [43, 44]. Each feature (EC relative abundances) was first scaled between 0 and 1 using the vegan package [45] and only features present in more than one-third of the samples were included in the XGBoost model. The dataset was then randomly split into training (80%) and test (20%) sets stratified by climate zone using the rsample package [46]. Models were run 25 times using independent splits to account for lucky and unlucky splits. For each run, model tuning was based on three-fold cross-validation of training data combined with Bayesian optimization [47] to select the best hyperparameters (eta, gamma, max_depth, min_child_weight, lambda, alpha) using AUC as the evaluation criteria. To compare accuracies of all model types, R^2^ values between observed versus predicted for all 25 models and all indicators were graphed as a distribution. A linear model between observed versus predicted values was generated for each model using the appropriate test datasets [48, 49]. R packages used for modeling include xgboost [50], caTools [51], and caret [52].

For each soil health indicator rating, the top enzymes were selected (e.g., enzymes present in 13 or more of the 25 independent models runs and ranked by their average gain across all model runs where the enzyme was present) to create a molecular index of soil health (MISH). Any enzyme (normalized relative abundance) that exhibited a negative Spearman correlation with the indicator of interest was first inverted (subtracted from 1) and then a weighted mean was calculated using the scaled relative abundances with average gains as weights to create individual MISH indicator ratings. An overall MISH rating was created similarly using enzymes from the previous step. The number of enzymes to select from each rating for the overall MISH rating was determined by creating the MISH score with variable numbers of enzymes (from 10–100), running a regression between the MISH indicator rating and the SEMWISE indicator rating, and observing when the R^2^ and average AIC reached a maximum. If a feature was common between two or more soil health indicators, the maximum gain was used for weighting.

The ability of the MISH ratings to capture differences associated with soil health indicators or management was assessed by comparing the distribution of ratings across indicator bins (very low: 0–20, low: 20–40, med: 40–60, high: 60–80, and very high: 80–100) using the non-parametric Kruskal-Wallis test with pairwise comparisons using Wilcoxon rank sum tests with FDR adjustment. All bins were constructed to account for climate and textural differences to enable comparisons across regions, soil types, and individual management practices.

The top enzymes for each indicator were assigned to KEGG pathways [53], which provides information on the function of each enzyme. All enzyme names and classifications were extracted from the ExplorEnz database, which is the approved International Union of Biochemistry and Molecular Biology Enzyme nomenclature and classification list [54].

## Results and discussion

### Data quality and coverage

**Fig 1:**
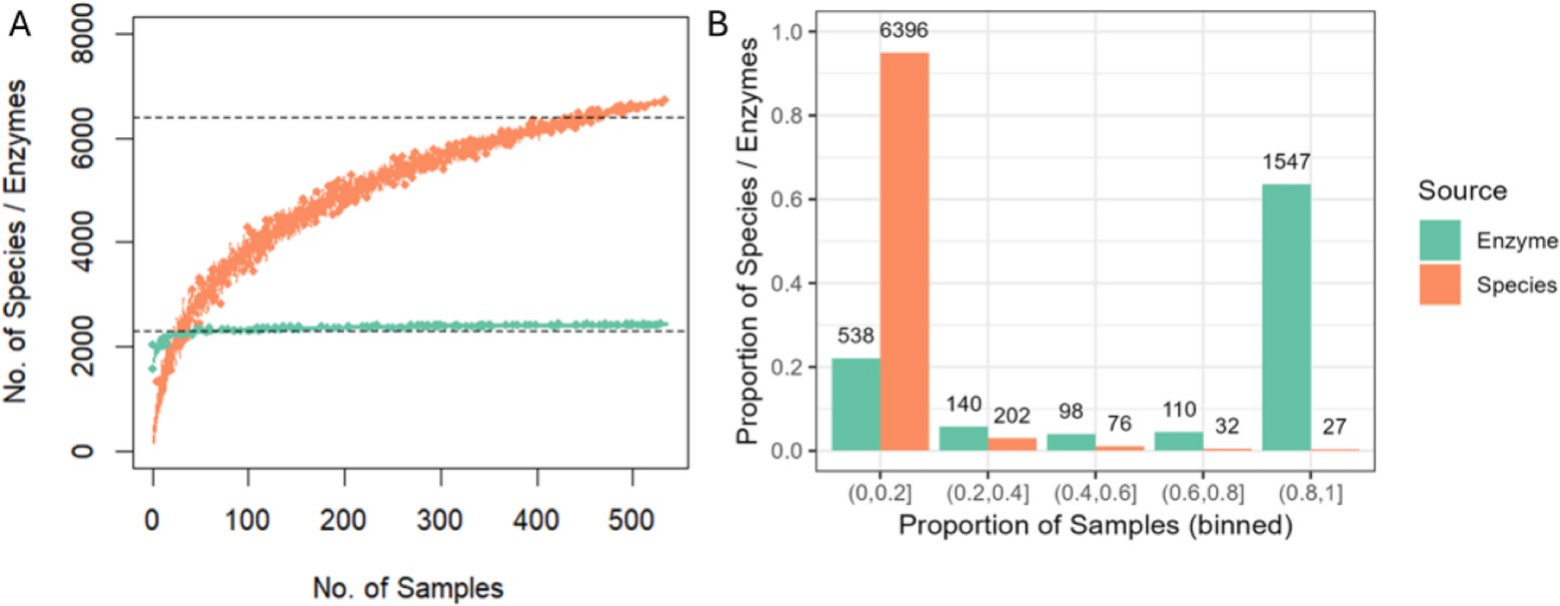
Collector’s curve and proportion analysis of species or enzyme prevalence. (A) Number of observed species (orange points) or enzymes (green points) as sample number increases. (B) Histogram of the proportion of species (orange bars) or enzymes (green bars) present in each sample, binned by the proportion of samples. Values above each bar are the number of species or enzymes in that bin.

A total of eight MiSeq runs were conducted resulting in 7,332,013 high-quality sequencing reads, approximately 2 Gbp of sequence data, and an average sequencing depth of 17,262 reads per sample. For the entire dataset, we observed a total of 6,733 bacterial species and a total of 2,433 bacterial enzymes after phylogenetic reconstruction with PICRUSt2. A collector’s curve analysis showed that approximately 450 and 50 samples were required to reach 95% coverage of the total taxonomic and enzymatic richness, respectively (Fig 1A). Furthermore, the enzyme collector’s curve flattened while the species curve did not, indicating that we had likely captured the full enzyme community but not species. In a typical sample, an average of 280 species and 1,704 enzymes were present, representing 4% and 70% of the total potential richness. In addition, there were 1,547 enzymes present in over 80% of samples, while most species were present in less than 20% of samples (Fig 1B).

Since species are often unique to smaller subsets of samples, as shown here, more samples are required to create an accurate model. Furthermore, it is uncertain whether unique species perform the same functions (i.e., functional redundancy). By using an enzyme approach, we circumvent the need to characterize functional redundancy, and we utilize the universally present enzymes to create a widely applicable model for measuring soil health. However, PICRUSt2 also has potential limitations. First, due to its DNA-based nature, it is not a direct measure of enzyme presence or activity but rather a measure of functional gene abundance or capacity. This limitation is true for any DNA-based marker gene or metagenome sequencing project as activity will ultimately depend upon gene expression and protein activity. Second, there is some uncertainty in applying a phylogenetic approach using a single marker gene (i.e., 16S rRNA) [55]; however, previous studies have shown that PICRUSt2 can be highly correlated with metagenome sequencing [29, 30], but for a fraction of the price. Overall, previous studies and the data shown in Fig 1 highlight the robustness of using enzymes to develop a soil health index due to its ubiquity across a variety of soils and its cost-effectiveness.

### Microbial functional data predicts soil health indicators

Since the goal is to develop a comprehensive index, we ran each model 25 times and compiled the results. This is because random forest variable importance measures are biased [56], and repeating the model increases confidence in the enzymes identified as important to soil health indicators. A compilation of the 25 models predicting soil health indicator ratings from the PICRUSt2 functional genes (enzyme relative abundances) was developed for each of the six soil health indicators. Linear regression p-values of predicted versus observed values for the test sets were significant (p < 0.001) for all soil health indicators. Mean adjusted R^2^ values for each soil health indicator rating ranged from 0.221 to 0.337 (Table 1) and root mean square errors (RMSE) ranged from 0.239 to 0.251 (Table 1). ACE protein (0.337) and SOM (0.310) measurements had the highest mean R^2^ values with WaterCap (0.221) and Resp (0.223) the lowest. For the 25 independent model runs, the average number of enzymes retained in the models ranged from a low of 359 (AggStab) to a high of 554 (SOM) (Table 1). There was significant variation in the number of enzymes retained in each random forest model depending on the train/test data split. For example, the number of enzymes ranged from 8 to 1164 for ACE protein (Table 1). The enzymes selected also varied between each model run with a range of 0–7 enzymes present in all 25 model runs for each indicator (S2 Fig).

**Table 1:** Summary statistics of XGBoost modeling results. Data included is from all 25 models and each soil health indicator. RMSE = root mean squared error. No. of Enzymes represents the number of enzymes retained in the model after feature selection.

	Adjusted R^2^		RMSE		No. of Enzymes				
Indicator	Mean	SD	Mean	SD	Mean	SD	Min	Max	p-value
ACE	0.337	0.077	0.239	0.019	451	310	8	1164	<0.001
ActiveC	0.262	0.080	0.248	0.015	413	245	8	894	<0.001
AggStab	0.270	0.064	0.251	0.015	359	246	13	987	<0.001
Resp	0.223	0.084	0.256	0.012	372	300	20	1163	<0.001
SOM	0.310	0.056	0.240	0.014	554	214	195	1133	<0.001
WaterCap	0.221	0.051	0.249	0.016	397	246	11	1085	<0.001

Due to the 25 model repetitions and the feature selection implemented by XGBoost, many enzymes were not present in all models. To compile these results, a list of potential “important” enzymes was created, including enzymes that were present in more than half of the model runs and had the highest impact on model accuracy or gain. These enzymes included both positively and negatively correlated with the indicators and may not necessarily be defined by linear relationships. Most enzymes were not included in the models, with an approximate range of 15–23% of the total number of identified enzymes included in any single model run.

**Fig 2:**
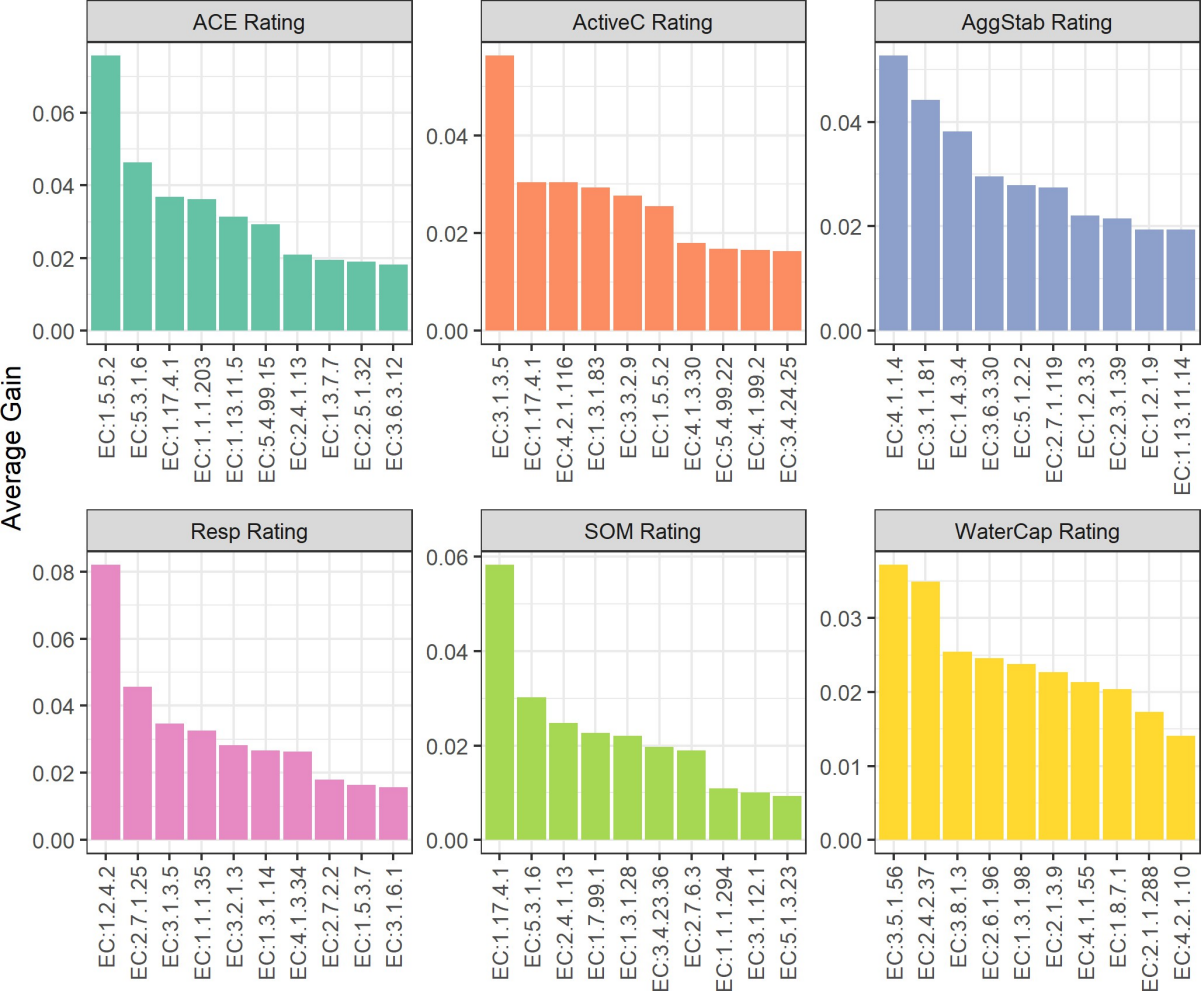
Enzymes with the top ten average gains for each soil health indicator rating. For each enzyme on the x-axis, the average gain on the y-axis was calculated by averaging the gains from all 25 XGBoost models for the respective indicator (see section Molecular index development and testing for method of gain calculation). Enzymes are labelled by their Enzyme Commission numbers and additional details about each enzyme can be found in S1 and S2 Tables.

**Fig 3:**
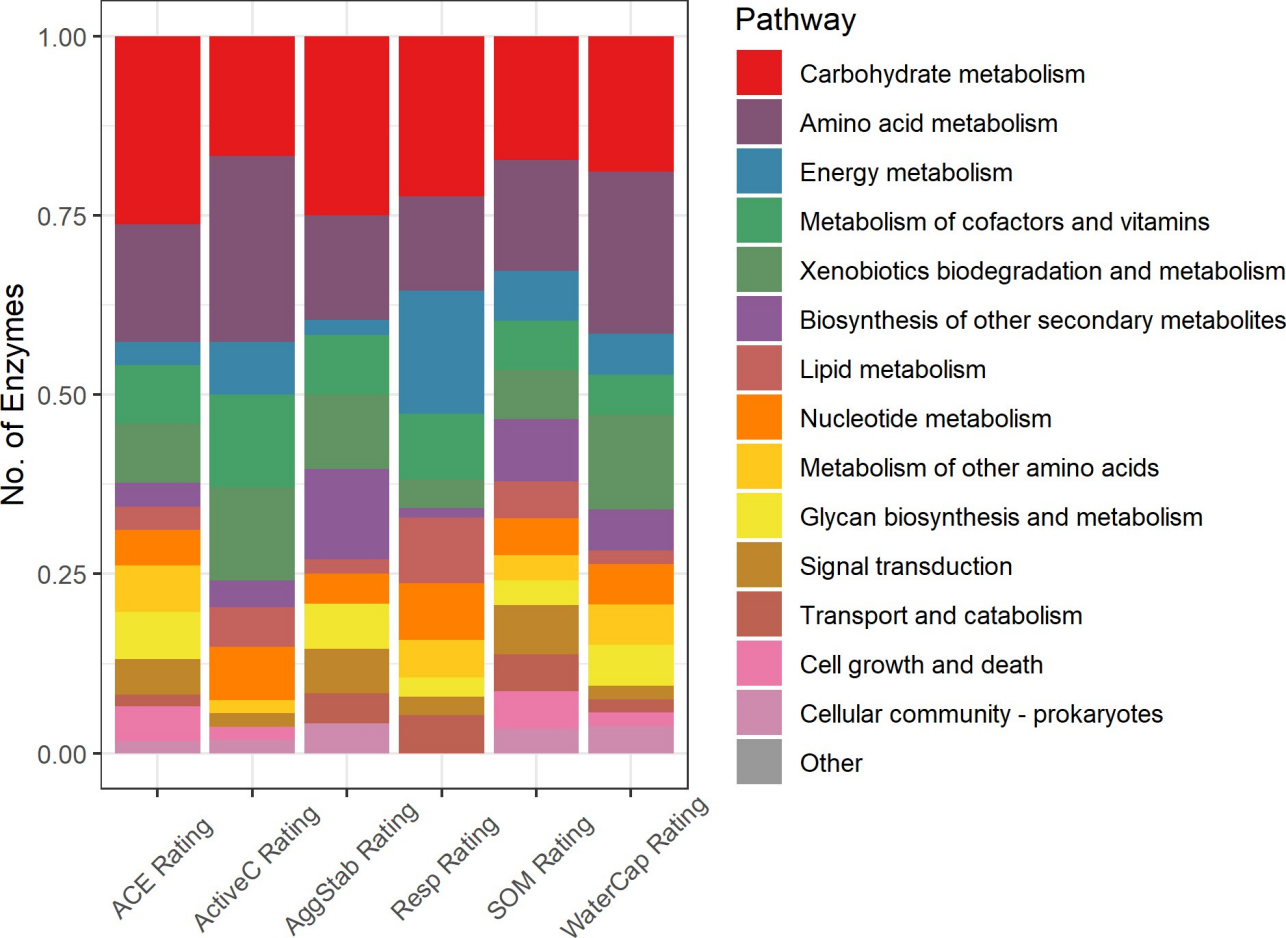
Number of enzymes associated with each KEGG pathway for each soil health indicator rating. Each enzyme may be classified to more than one pathway and the reported values are expressed as a proportion of the total classifications. The “Other” pathway represents pathways that were irrelevant to enzyme function in soils (i.e., pathways that were human-related).

The enzymes with the top ten average gains for each soil health indicator tended to be unique for each indicator (Fig 2 and S1 Table), and they spanned a range of KEGG pathways (Fig 3 and S2 Table) and enzyme classes (S3 Table). The top 50 enzymes for each indicator were compiled. Since some of the top enzymes were common between indicators, this resulted in a final list of 235 unique enzymes. Of these 235 enzymes, 22.6% were associated with “Carbohydrate metabolism”, 20.9% with “Amino acid metabolism”, 9.8% with “Energy Metabolism” or “Metabolism of cofactors and vitamins”, 9.4% with “Xenobiotics biodegradation and metabolism”, 6.8% with “Biosynthesis of other secondary metabolites”, and 6.4% with “Lipid metabolism” or “Nucleotide metabolism”; all other KEGG pathways were associated with less than 5% of the enzymes. For each indicator, the distribution of enzymes and their mapped KEGG pathways differed (Fig 3 and S2 Table). The pathways represented by the 50 important enzymes for the SOM rating was most diverse (Fig 3), likely because organic matter is complex and requires the use of many bacterial enzymes to break it down.

Carbohydrate metabolism was among the most common pathway for all six indicators. Many of these enzymes (a full list is shown in S2 Table) are dehydrogenases, which are known to oxidize SOM as part of the microbial respiration pathway [25]. This may explain why the carbohydrate metabolism pathway, closely followed by energy metabolism, have the highest proportion of enzymes in the Resp rating (Fig 3). Carbohydrate metabolism was also the most abundant pathway in the ACE and AggStab ratings (Fig 3).

Amino acid metabolism was the next most abundant pathway of the top enzymes. Manipulating amino acid metabolism has been shown to improve crop nitrogen (N) use efficiency through regulating N uptake, assimilation, and remobilization efficiencies [57]. Amino acids are a key mobilizable source of N for plants in which the N is made available by extracellular microbial enzymes through deamination and the release of ammonium N [58]. This influx of N can then influence soil aggregation, either by increasing [59] or decreasing [60] its stability. Additionally, amino acid metabolism has been shown to maintain energetic balance by coordinating with carbohydrate metabolism [61], the most abundant pathway. Amino acid metabolism had the highest relative abundance in the ActiveC and WaterCap ratings (Fig 3). Active C has been shown to be associated with soil N availability [62, 63], and a low C:N ratio is needed to store and maintain N in the soil organic matter [41]. Water availability has been shown to affect amino acid composition [64], and the associated enzymes identified here may be targeted in future studies to better understand the role that microbes play in this relationship.

Two enzymes that were within the top predictive enzymes of several indicator ratings are notable. EC 1.5.5.2, a proline dehydrogenase involved in amino acid metabolism, increased with ACE, Active C, SOM, and WaterCap ratings (S1 Table). Additionally, EC 1.17.4.1, a reductase involved in DNA repair, significantly increased with ACE, Active C, and SOM ratings (S1 Table). Both enzymes are likely constitutive, or always present in the soil [25] and have consequently been ignored in studies relating microbial enzymes and soil health. However, their positive correlation with several soil health indicators warrants further investigation. The top enzymes showed both positive and negative correlations with the soil health indicators (S1 Table). It is beyond the scope of this paper to determine if the positive enzymes are responsible for building soil health or responding to the higher levels of C and N (e.g., SOM) increasing microbial growth and survival and thus enzyme abundances. However, these enzymes are the most consistent and important features for predicting the various soil health indicators and may be key for developing indices for predicting soil health from a single low-cost 16S rRNA amplicon analysis.

### Molecular index of soil health

Rather than supplying a single machine learning model as the tool for measuring soil health, we chose to develop a comprehensive molecular index that incorporated results from multiple (25) models. Random forest variable importance measures are biased [56] such that the split during tree generation can change which features are identified as most important. By running 25 models, our goal was to account for this bias and identify enzymes that are consistently important to soil health. These results could then be combined into a final, simplified index that includes few, but important, enzymes, and still has accurate prediction of soil health. This would additionally allow for the index to be readily applied across other datasets.

To compile important enzymes into a molecular index of soil health (MISH), the optimal number of enzymes to incorporate was first selected using average R^2^ and Akaike Information Criterion (AIC). Although all regressions between the MISH indicator rating and SEMWISE indicator ratings were significant, average R^2^ and AIC appeared to reach a maximum at 50 enzymes (S3 Fig). We chose to create individual indicator indices as well as an overall MISH index to determine which is more predictive across a variety of climates. Therefore, the top 50 important enzymes from each soil health indicator rating were compiled into individual indicator ratings and an overall MISH rating in which all enzymes were combined, resulting in a total of 235 unique enzymes.

**Fig 4:**
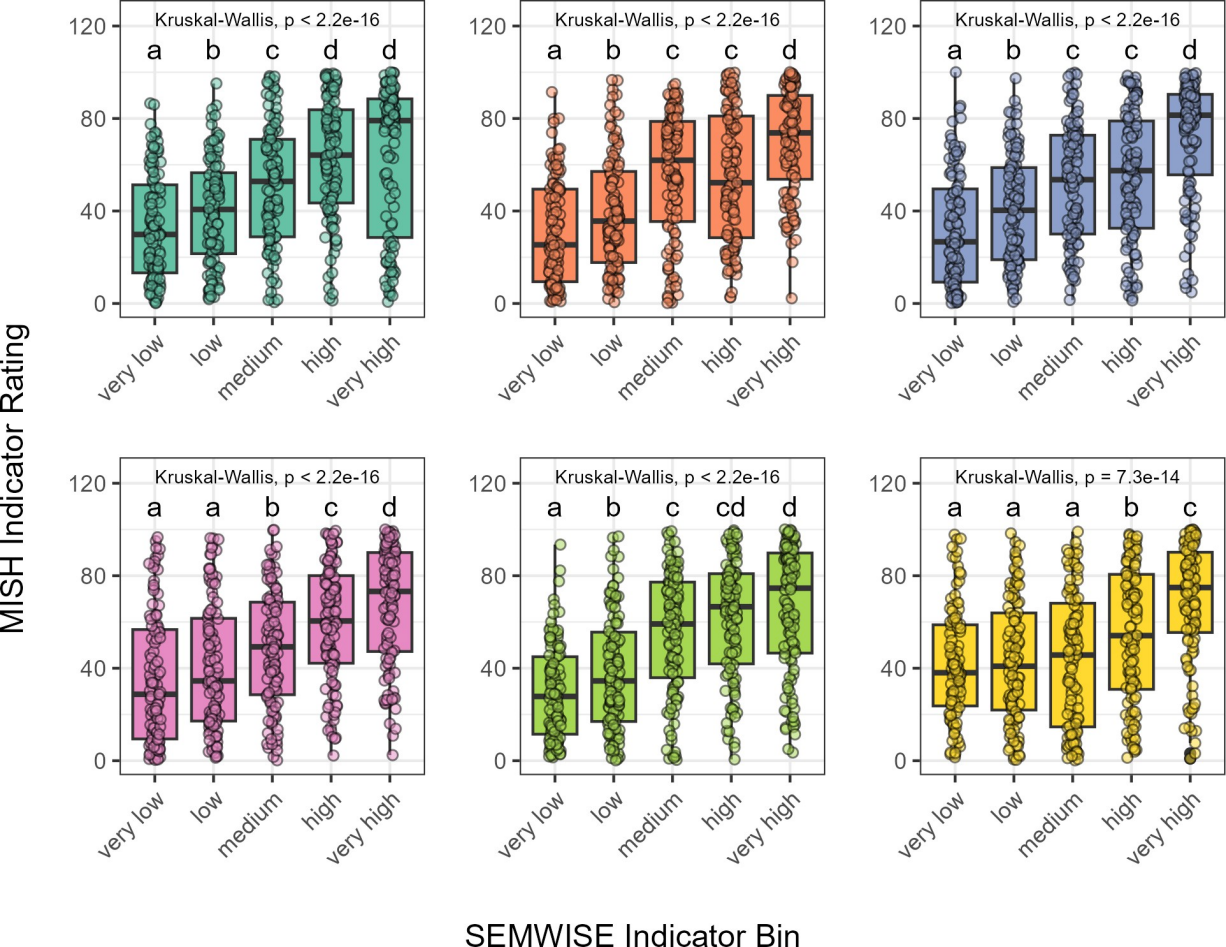
MISH indicator ratings between SEMWISE indicator bins for each soil health indicator. SEMWISE Indicator Bins represent the individual indicator score in which “very low” is the lowest soil health and “very high” is the highest soil health based on the SEMWISE model described in Deel et al. [34]. The MISH Indicator Ratings were calculated using the top enzymes for each indicator (see section Molecular index development and testing for calculation) and are expressed as a percentage between 0–100. Different letter labels indicate a significant difference between bins.

For all six SEMWISE indicator ratings, MISH indicator ratings were significantly different between bins based on a non-parametric Kruskal-Wallis test (p < 0.001) (Fig 4). MISH indicator ratings tended to significantly increase (p < 0.05) with successive SEMWISE indicator bins based on Wilcoxon rank sum tests with FDR adjustment. For each indicator, a single MISH indicator rating derived from only 50 commonly occurring enzymes was sufficient to predict the six soil health indicators from a wide range of agricultural systems across the U.S.

**Fig 5:**
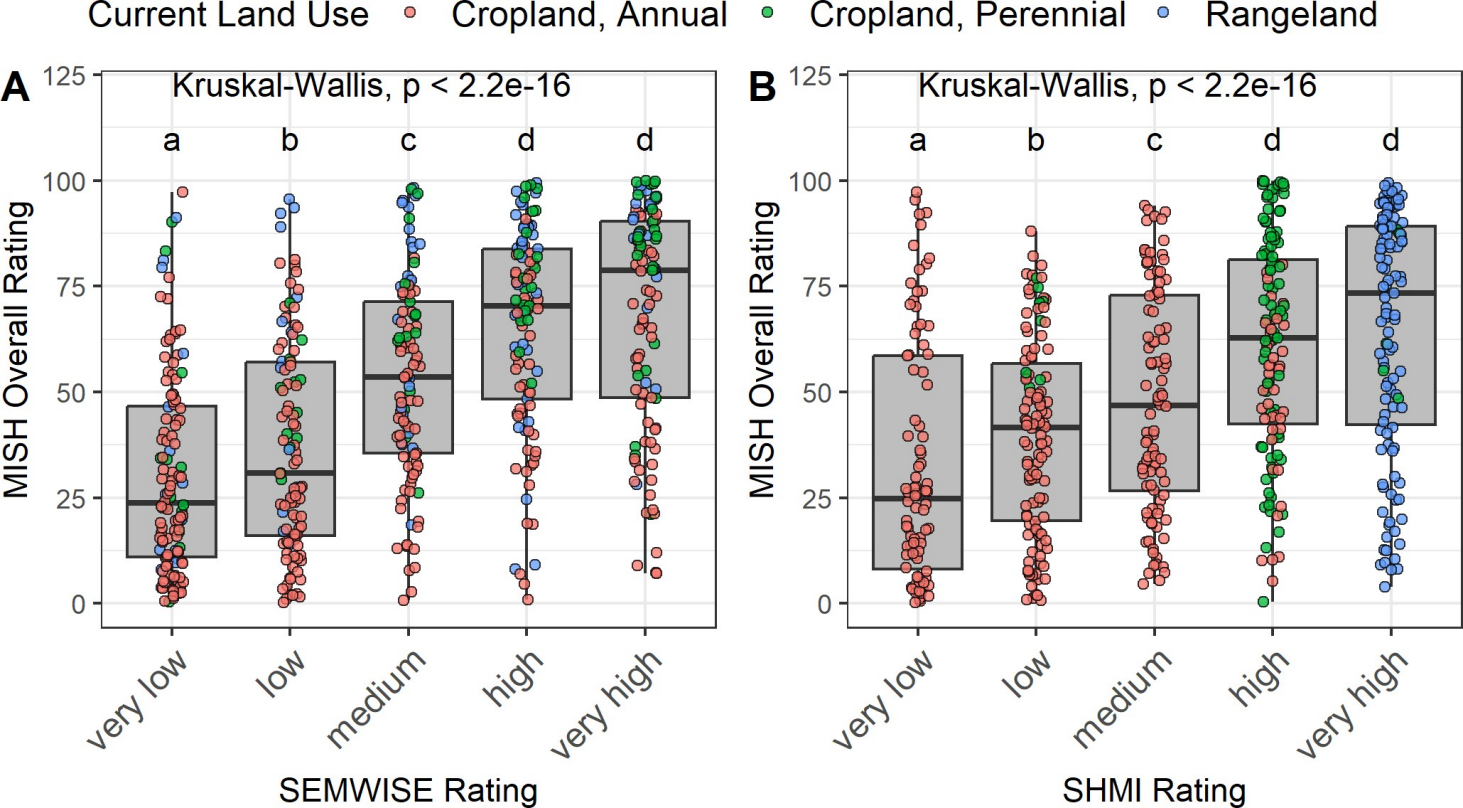
MISH Overall Rating vs SEMWISE and SHMI rating bins. Each panel shows the distribution of MISH Overall Ratings binned by (A) soil health (SEMWISE) rating or (B) management (SHMI rating). Both ratings on the x-axes are described in Deel et al. [35]. Briefly, the SEMWISE Rating represents the soil health score that incorporates all soil health indicators. The SHMI rating represents the overall management score based on the soil health principles. In both ratings, the “very low” bin represents the lowest soil health and “very high” bin represents the highest soil health. The MISH Overall Rating was calculated using top enzymes from all indicators (see section Molecular index development and testing for calculation) and is expressed as a percentage between 0–100. Different letter labels indicate a significant difference between bins. Points are color-coded by current land use with annual cropland (red), perennial cropland (green), and rangeland (blue).

Similar to the individual ratings, we compared the MISH overall rating to binned SEMWISE ratings (Fig 5A). The MISH overall ratings were significantly higher with each successive SEMWISE bin in the very low, low, med, and high categories but not the very high bin. One of the difficulties in conducting national-scale assessments of soil health is due to differing combinations of management practices that may co-exist in time and space. For example, two sites may both have cover crops but one is under no-till and the other conventional tillage and/or sites may differ in the diversity of crops. This complexity makes it difficult to compare ratings across sites. To address these complexities, we previously introduced a soil health management index (SHMI) that combines soil health management practices into a single index [34]. The SHMI bins represent combinations of practices that manage soil health through the principles to minimize soil disturbance, increase plant diversity, and provide continuous soil cover and living roots [34]. This binning procedure resulted in different land uses typically assigned to specific bins. For example, the very high bin is represented by rangeland, perennial cropland dominated in the high bin, and annual cropland was spread among the very low to high bins (Fig 5). Typical annual cropland management systems for each SHMI bin are as follows: conventionally tilled, monoculture cropping systems (very low); no-till monoculture cropping systems (low); conventionally tilled with cover crops or diversified crop rotations (medium); and no-till plus cover crops and/or diversified crop rotations (high). The SHMI scores were calculated based on a two- to three-year management history and some of the samples experienced a transition in management over that period (i.e., annual cropland converted to perennial cropland or green dots in the low SHMI bin), which resulted in some of the inconsistency in SHMI rankings across land use types. Across the entire national dataset, MISH overall ratings significantly increased (p ≤ 0.05) with each successive SHMI bin, except the very high bin (Fig 5B). The overall congruence between the two measures suggests that as soil health management practice adoption increases, the MISH overall score increases. This trend is similar to those seen for the individual indicator ratings (Fig 4), suggesting that individual indicator ratings are not more accurate, and an overall index is suitable for comparison across locations. In its current form, the SHMI rating does not address the length of time a management practice has been in place. Soil health indicators can vary in their response times to management changes. For example, converting from conventional to no-tillage can take up to 20 years to reach a new SOM equilibrium [65]. Efforts are currently underway to improve our SHMI rating to account for time, which should further improve the relationship between MISH and SHMI at the national scale.

## Conclusions

In this study, we used PICRUSt2 to estimate enzyme or functional gene relative abundances and developed individual scores and an overall molecular index of soil health (MISH). Enzymes were first selected using XGBoost modeling to identify the most important enzymes for predicting known soil health indicators (ACE, ActiveC, AggStab, Resp, SOM, and WaterCap). From these models, individual MISH ratings were constructed for each indicator, as well as an overall MISH rating from the most important enzymes associated with each of the six indicators. The individual MISH indices were positively correlated and showed good agreement with the soil health indices across the 536 samples from this national assessment of U.S. agricultural systems. An overall MISH index was also positively correlated with overall measures of soil health (SEMWISE) and management practices (SHMI). Additionally, since the MISH index was created using indicator data that was corrected for clay content and climate zone and based on enzymes present in all samples, it is suitable across multiple regions and agricultural systems. By leveraging the power of phylogenetic reconstruction using PICRUSt2, this assay involves a single 16S rRNA amplicon sequencing approach that is relatively low cost and easily employed in molecular biology laboratories. This new, molecular-based index correlates with soil health indicators and management. It is a quick, easy, and inexpensive way to measure and compare microbial contributions to soil health, and will be particularly useful for surveys, meta-analyses, and long-term studies.

## Supporting information

S1 Fig Construction of SEMWISE indicator ratings. (TIF)

S2 Fig Number of common enzymes in the number of models for each soil health indicator. For example, for the ACE rating, in two models there ~1600 common enzymes retained in both models. In ten models, there were less than 400 retained enzymes in common between all ten models.
(TIF)

S3 Fig Criteria for selecting the number of enzymes in each MISH indicator rating. No. of enzymes = the number of enzymes selected (based on presence in >13 of the random forest models and highest average gain) and used to create the MISH score. Adjusted R^2^ and average Akaike Information Criteria were calculated from a regression between the MISH indicator rating with the selected number of enzymes and the SEMWISE indicator rating.
(TIF)

S1 Table Enzymes with the top 50 average highest gains for each soil health indicator. (XLSX)

S2 Table The KEGG pathways of all enzymes with high gains. (XLSX)

S3 Table The enzyme classes of all enzymes with high gains. (XLSX)
