# Supplementary figures and images for: Rethreading the needle: A novel molecular index of soil health (MISH) using microbial functional genes to predict soil health management
Source: PLoS One. 2024 Dec 2;19(12):e0314072. doi: 10.1371/journal.pone.0314072 (PMC11611206; doi:10.1371/journal.pone.0314072)

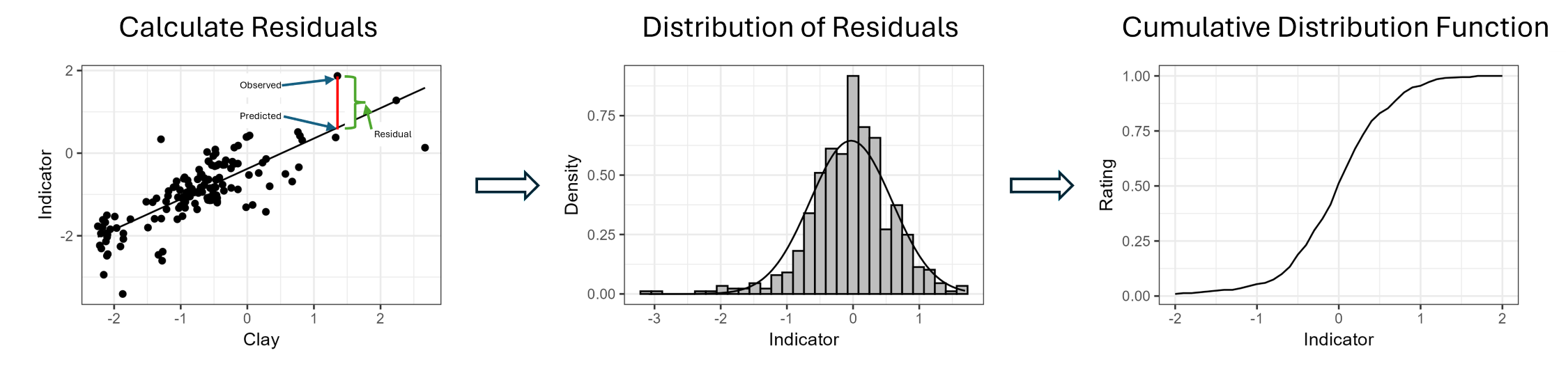

Supplement: S1 Fig — (TIF) [file pone.0314072.s001.tif]

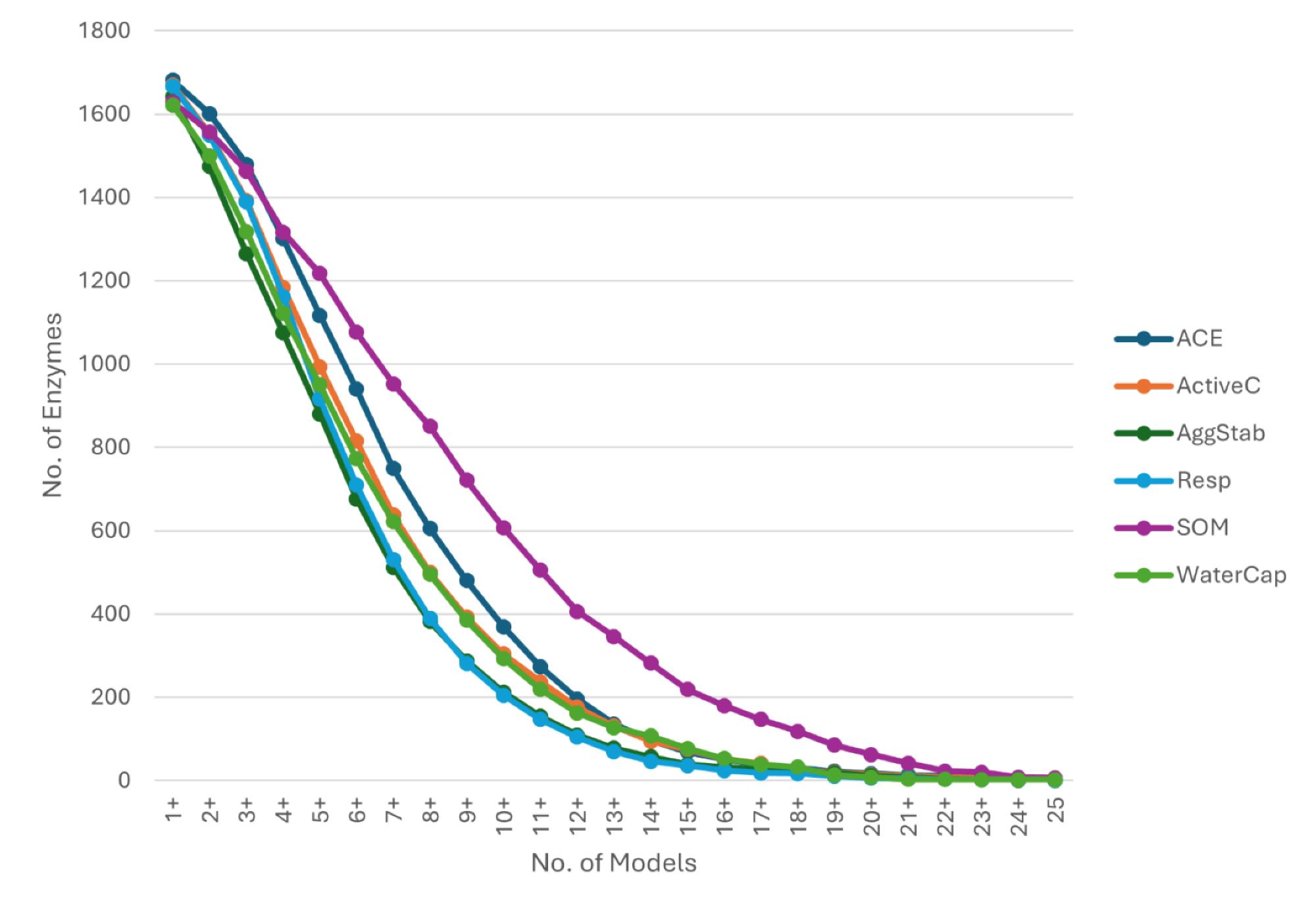

Supplement: S2 Fig — For example, for the ACE rating, in two models there ~1600 common enzymes retained in both models. In ten models, there were less than 400 retained enzymes in common between all ten models. (TIF) [file pone.0314072.s002.tif]

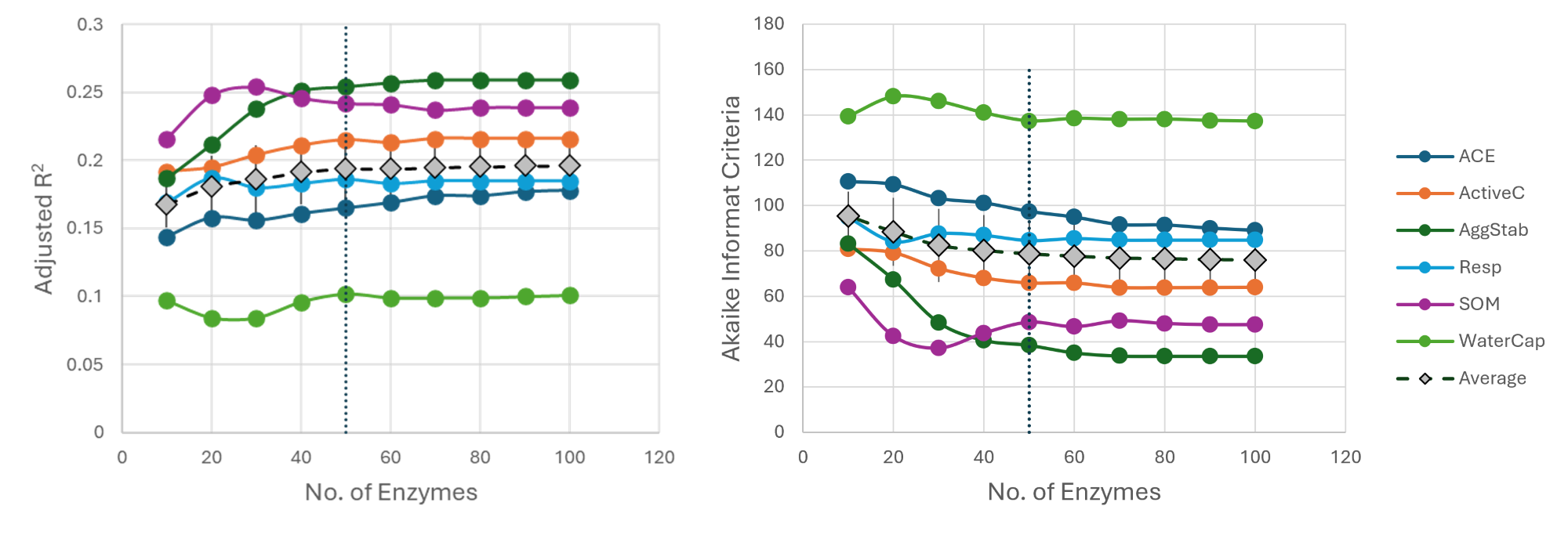

Supplement: S3 Fig — No. of enzymes = the number of enzymes selected (based on presence in >13 of the random forest models and highest average gain) and used to create the MISH score. Adjusted R2 and average Akaike Information Criteria were calculated from a regression between the MISH indicator rating with the selected number of enzymes and the SEMWISE indicator rating. (TIF) [file pone.0314072.s003.tif]
